# Production of Volatile Compounds in Reconstituted Milk Reduced-Fat Cheese and the Physicochemical Properties as Affected by Exopolysaccharide-Producing Strain

**DOI:** 10.3390/molecules171214393

**Published:** 2012-12-05

**Authors:** Weijun Wang, Lanwei Zhang, Yanhua Li

**Affiliations:** School of Food Science and Engineering, Harbin Institute of Technology, Harbin 150090, China; E-Mails: wangweijunid@yahoo.com.cn (W.W.); liyanhua607@sohu.com (Y.L.)

**Keywords:** volatile compounds, exopolysaccharide, reconstituted milk, cheese

## Abstract

The application of the exopolysaccharide-producing strains for improving the texture and technical properties of reduced-fat cheese looks very promising. *Streptococcus thermophilus* TM11 was evaluated for production of reduced-fat cheese using reconstituted milk powder (CRMP). The physicochemical analysis of fresh and stored cheeses showed that this strain slightly increased moisture content resulting in cheese with higher yield and lower protein content compared to the direct acidified cheese. The volatiles of cheese were determined by SPME and GC equipped with a mass spectrometer. The results indicated that the major compounds included aldehydes, ketones and acids, whereas, alcohols and branched-chain aldehydes that contribute to exciting and harsh flavors were not found in CRMP. By the textural profile analysis, we found the cheese made with *S. thermophilus* TM11 had lower cohesiveness, resilience and higher adhesiveness than the direct acidified cheese, and had similar hardness. Further, *S. thermophilus* TM11 greatly changed the protein matrix with more opened cavities according to observation by scanning electron microscopy. Consequently, use of *S. thermophilus* TM11 could endow CRMP with the novel and suitable flavor properties and improved texture quality.

## 1. Introduction

Flavor and texture of a food are critical factors in both product evaluation and consumer acceptability. For decades, cheese consumption was stagnating in China. The sensory characteristics of cheese seem to be the main obstacles. Because cheese, like yoghurt, is a non-traditional food brought from the West into China, many consumers do not like this food due to its strong flavor. However, yoghurt has been very successful in achieving high acceptability and high output in China. In 2005, over 500,000 tons of yoghurt were produced [1], while, the cheese consumption in 2006 was only 20,000 tons, of which 90% were imported [2], mainly as materials for pizza processing and production of processed cheese. 

The dietary culture and preferences of the Chinese are significantly different from those of Western people [3], so the major focus of cheese development in China should be focused on the short ripened, high value and well accepted soft or semi-soft cheeses [1]. The flavors of “sour” and “milky” with “slimy” and “moist” textures appeared to be drivers of liking for most Chinese youth, while the flavors of “bitter”, “salty” and “free fatty acid” with “firmness” texture were drivers of disliking [3].

Lactose, protein and lipid catabolism are the main sources of aroma compounds in cheese. These pathways are activated by endogenous enzymes in milk, coagulating enzymes, and microbial enzymes used to manufacture or ripen cheese [4]. Among these factors, starter organisms play an important role, and can be used as an approach to improve or control the sensory quality. Starter organisms can contribute to proteolysis, and hence texture and flavor development [5]. Most studies were concentrated on the effect of selected starter cultures, e.g., the use of adjunct cultures [6] and exopolysaccharide (EPS)-producing starters [7]. Furthermore, many culture companies now provide lactic acid starter cultures that have been specially designed for the manufacture of different fermented milk products [8].

EPS produced by starter cultures are heteropolysaccharides. Perry *et al.* [9] found that EPS-producing *Streptococcus thermophilus* could increase moisture retention in low-fat Mozzarella cheese. EPS were responsible for the water-binding properties of this bacterium in cheese [10]. It was also confirmed that encapsulated and ropy EPS-producing *S. thermophilus* strains can be utilized to increase the moisture level of cheese. However, only the encapsulated EPS can improve these properties without adversely affecting whey viscosity [11,12].

Some other EPS-producing strains, for example *Lactococcus lactis* ssp. *cremoris* also could contribute to the modification of cheese texture, microstructure [13] and melting properties [14]. These and similar researches devoted their efforts to low fat cheeses because such cheeses tend to become tough, rubbery, and have poor stretching properties [15]. 

Similar deficiencies exist in the cheeses produced from reconstituted milk powder (CRMP). According to Tamime *et al*. [16], the experimental processed cheeses (made from Cheddar cheese and a cheese base produced from reconstituted dried skim milk) were markedly firmer than control cheeses (made from Cheddar cheese without cheese base). However, reconstituted dried milk has found applications in some soft cheese varieties. Leclercq-Perlat and coworkers introduced a soft smear cheese made from reconstituted skim milk powder using microorganisms to control the production [17,18]. As far as we known, no research has been devoted to the application of EPS-producing starter cultures in CRMP, although these cultures have been widely used in the production of yoghurt from reconstituted milk powder [19,20].

Because of the economic importance and recent practice of producing reconstituted milk cheeses, this research was focused on the identification of volatile compounds that contribute to the flavor and aroma in CRMP. Additionally, the effect of EPS on overall flavor and texture of this cheese was considered. *S. thermophilus* TM11 and SP1.1 were commercial strains which were isolated from Chinese traditional fermented milk by our lab. These strains have been widely used for yoghurt production in China. *S. thermophilus* TM11 is a good EPS-producing strain. The application of these starter cultures for the development of new cheeses and/or flavors looks very promising.

## 2. Results and Discussion

### 2.1. Physicochemical Analysis

The physicochemical analysis of CRMP is illustrated in Table 1. In general, *S. thermophilus* TM11 led to a slight increase in cheese yield by 4.6%. This minor increase was due to the production of EPS. Similar results were reported by Petersen *et al*. [12] in Mozzarella cheese, by Dabour *et al.* [13] in Cheddar cheese, and by Jimenez-Guzman *et al.* [21] in Panela cheese. On contrary to the direct acidified cheese (DAC), the effect of *S. thermophilus* SP1.1 on the yield was insignificant (*p* > 0.05).

**Table 1:** Yield, protein, salt, moisture, pH and lactococci counts of CRMP.

Samples	Yield (%)	Protein (%)	Salt (%)	Moisture (%)	pH	Lactococci counts [LogCFU/g]
Direct-acidified cheese						
Day 1	23.7 ± 0.3 ^a^	25.6 ± 0.4 ^a^	1.71 ± 0.08 ^a^	65.0 ± 0.8 ^ab^	6.32 ± 0.04 ^a^	-
Day 21	-	-	-	62.5 ± 0.9 ^cd^	6.28 ± 0.05 ^a^	-
Day 45	-	-	-	59.9 ± 1.3 ^e^	6.29 ± 0.06 ^a^	-
Cheese with *S. thermophilus* TM11						
Day 1	24.8 ± 0.4 ^b^	23.9 ± 0.4 ^b^	1.73 ± 0.12 ^a^	66.5 ± 0.7 ^a^	5.13 ± 0.11 ^b^	7.7 ± 0.2 ^a^
Day 21	-	-	-	66.3 ± 0.5 ^a^	5.08 ± 0.13 ^b^	7.8 ± 0.2 ^a^
Day 45	-	-	-	65.9 ± 1.1 ^a^	5.01 ± 0.16 ^bc^	7.5 ± 0.3 ^a^
Cheese with *S. thermophilus* SP1.1						
Day 1	23.5 ± 0.3 ^a^	25.3 ± 0.3 ^a^	1.74 ± 0.08^a^	64.8 ± 0.7 ^abc^	4.98 ± 0.03 ^bc^	8.7 ± 0.3 ^b^
Day 21	-	-	-	63.1 ± 0.6 ^bcd^	4.89 ± 0.03 ^bc^	8.7 ± 0.2 ^b^
Day 45	-	-	-	62.1 ± 0.7 ^de^	4.80 ± 0.06 ^c^	8.6 ± 0.3 ^b^

CFU, colony-forming unit; ^a–e^ Means ± SD, from three replicate cheese-making trials, in each column with different letters were significantly different (*p* < 0.05).

Moisture of DAC decreased during storage (*p* < 0.05). Meanwhile, the cheese made with *S. thermophilus* TM11 showed no change in moisture, and the cheese made with *S. thermophilus* SP1.1 showed a slight decrease. On days 21 and 45, compared to cheese made with *S. thermophilus* TM11, DAC significantly decreased (*p* < 0.05) cheese moisture by 5.7% and 9.1%, respectively. The results suggested that EPS-producing *S. thermophilus* TM11 had a strong capacity of moisture retention and could be used in CRMP to solve the problem of whey leakage during storage.

CRMP produced by *S. thermophilus* TM11 had a slightly lower protein content than other cheeses (*p* < 0.05). The increased moisture retention was the main reason, although the drain processing also caused the loss of total protein. The protein percentage (%), calculated as the ratio of the protein content to the total solids content, had no difference among the cheeses: 41.6% for TM11 cheese, 41.8% for SP1.1 cheese and 42.3% for DAC (*p* > 0.05).

*S. thermophilus* SP1.1 has a higher capacity to proliferate and acidify than *S. thermophilus* TM11. CRMP made with *S. thermophilus* SP1.1 had higher lactococci counts and lower pH value than that of *S. thermophilus* TM11. The difference in strain counts may be due to the continuous proliferation during the cooling of cheese made with strain SP1.1, or due to the loss of bacteria with whey in drain processing of cheese made with strain TM11. The pH of cheese made with *S. thermophilus* SP1.1 decreased slightly during storage, and a statistically significance was found between the pH on days 1 and 45 (*p* < 0.05).

### 2.2. Volatile Compounds in CRMP

**Figure 1:**
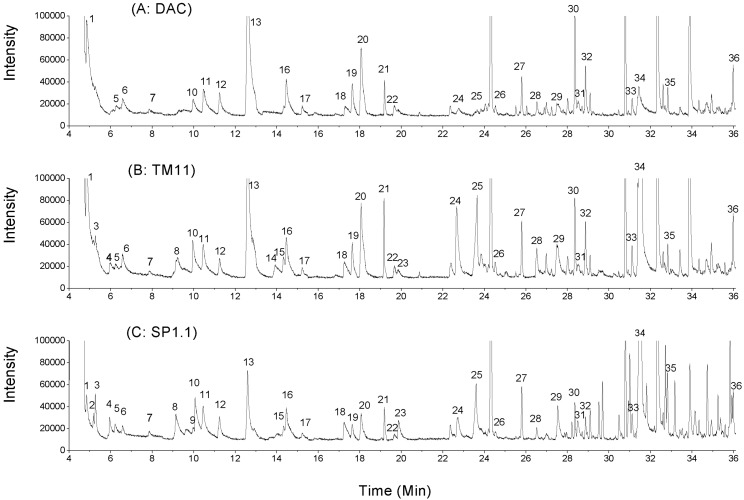
Comparison of typical total ion currency (TIC) profile of the volatiles from the direct-acidified cheese (**A**: DAC) and the cultured cheeses with *S. thermophilus* (**B**: TM11 and **C**: SP1.1). Peak numbers, 1–36, were marked. Alkanes, heterocyclic compounds excluding indene, pollutants (such as naphthalene, 32.34 min) and the un-identified chemicals were not listed.

In total, 35 aroma compounds were evaluated in CRMP (Figure 1 and Table 2). These compounds belong to different chemical families, including aldehydes, ketones, esters, acids, sulfur compounds, aromatic compounds and oximes. Note that alkanes, pollutants and unidentified chemicals were not listed. Table 2 shows the aroma compounds and their relative contents (AR values) determined in CRMP on the day 45 of storage. 2,3-Butanedione, 2,3-pentanedione, 3-hydroxy-2-butanone (acetoin), butyric acid and methoxyphenyl oxime were not observed in DAC, and acetoin was not detected in the cheese made from *S. thermophilus* TM11.

**Table 2:** Comparison of volatiles in the direct-acidified cheese (DAC) and the cultured cheeses with *S. thermophilus* (TM11 and SP1.1) on the day 45 of aging.

Peak number *	Retention time, min	Volatiles	AR		
			DAC	TM11	SP1.1
1	4.84	Acetone	5.80 ± 1.46 ^a ^	7.24 ± 3.57 ^a^	2.30 ± 0.94 ^b^
2	5.17	Thiourea	0.41 ± 0.35 ^a^	0.39 ± 0.47 ^a^	0.59 ± 0.27 ^a^
3	5.26	Carbon disulfide	0.79 ± 0.83 ^a^	1.29 ± 0.77 ^a^	1.67 ± 0.85 ^a^
4	5.95	2,3-Butanedione	-	1.02 ± 0.41 ^a^	0.84 ± 0.29 ^a^
5	6.22	2-Butanone	0.56 ± 0.68 ^a^	0.42 ± 0.57 ^a^	0.62 ± 0.81 ^a^
6	6.58	Ethyl acetate	1.11 ± 0.89 ^a^	1.66 ± 1.41 ^a^	0.54 ± 0.33 ^a^
7	7.85	Benzene	0.03 ± 0.02 ^a^	0.04 ± 0.03 ^a^	0.08 ± 0.04 ^a^
8	9.15	2,3-Pentanedione	-	3.58 ± 1.83 ^a^	2.34 ± 1.06 ^a^
9	9.99	3-Hydroxy-2-butanone	-	-	0.36 ± 0.22
10	10.07	Methyl 2-methyl-2-propenoate	1.17 ± 1.22 ^a^	2.03 ± 1.14 ^a^	2.50 ± 2.19 ^a^
11	10.45	Methyl butyrate	2.78 ± 0.99 ^a^	3.12 ± 0.80 ^a^	2.59 ± 1.25 ^a^
12	11.24	4-Methyl-2-pentanone	IS	IS	IS
13	12.79	Toluene	9.92 ± 3.57 ^a^	16.76 ± 4.80 ^b^	7.71 ± 4.48 ^a^
14	13.98	Butyric acid	-	1.52 ± 1.46 ^a^	0.05 ± 0.04 ^b^
15	14.33	Hexanal	0.15 ± 0.24 ^a^	0.93 ± 0.36 ^b^	0.34 ± 0.28 ^a^
16	14.46	Ethyl butyrate	4.97 ± 3.43 ^a^	6.12 ± 3.49 ^a^	3.69 ± 1.59 ^a^
17	15.22	Butyl acetate	0.97 ± 0.51 ^a^	0.70 ± 0.26 ^a^	0.58 ± 0.21 ^a^
18	17.25	2-Hexenal	0.59 ± 0.22 ^a^	1.70 ± 0.64 ^b^	0.51 ± 0.45 ^a^
19	17.63	Ethylbenzene	1.62 ± 0.84 ^a^	1.85 ± 0.51 ^a^	0.42 ± 0.36 ^b^
20	18.07	Xylene	4.64 ± 2.95 ^a^	5.42 ± 2.44 ^a^	1.22 ± 1.93 ^b^
21	19.16	2-Heptanone	1.82 ± 1.36 ^a^	2.45 ± 0.86 ^a^	0.57 ± 0.34 ^b^
22	19.68	Heptanal	0.77 ± 0.48 ^a^	0.54 ± 0.23 ^a^	0.14 ± 0.17 ^b^
23	19.94	Methoxy-phenyl-oxime	-	0.26 ± 0.14 ^a^	0.92 ± 0.56 ^b^
24	22.71	Benzaldehyde	1.13 ± 1.93 ^a^	6.00 ± 2.09 ^b^	1.22 ± 0.54 ^a^
25	23.61	Hexanoic acid	0.31 ± 0.15 ^a^	8.35 ± 3.55 ^b^	3.10 ± 1.43 ^c^
26	24.54	Octanal	0.57 ± 0.20 ^a^	0.92 ± 0.39 ^b^	0.20 ± 0.14 ^a^
27	25.80	Limonene	1.22 ± 0.53 ^a^	1.37 ± 0.38 ^a^	1.26 ± 0.76 ^a^
28	26.52	Indene	0.91 ± 0.36 ^a^	1.21 ± 0.42 ^a^	0.41 ± 0.32 ^b^
29	27.53	Acetophenone	0.43 ± 0.29 ^a^	2.70 ± 1.20 ^b^	1.48 ± 0.68 ^ab^
30	28.36	2-Nonanone	2.46 ± 1.38 ^a^	2.91 ± 1.25 ^a^	0.88 ± 0.62 ^b^
31	28.50	3,5-Octadien-2-one	0.51 ± 0.43 ^a^	0.65 ± 0.31 ^a^	0.41 ± 0.27 ^a^
32	28.87	Nonanal	1.94 ± 0.84 ^a^	1.85 ± 0.76 ^a^	0.57 ± 0.13 ^b^
33	31.12	2-Nonenal	1.15 ± 0.80 ^a^	1.83 ± 1.09 ^a^	0.79 ± 0.85 ^a^
34	31.47	Octanoic acid	4.40 ± 2.29 ^a^	17.97 ± 5.92 ^b^	14.23 ± 4.88 ^b^
35	32.81	Decanal	1.40 ± 1.37 ^a^	1.72 ± 0.79 ^a^	1.84 ± 1.04 ^a^
36	35.98	2-Undecanone	1.34 ± 0.78 ^a^	1.80 ± 0.74 ^a^	1.09 ± 0.51 ^a^

* Peak numbers were in conformity with the numbers marked in Figure 1; ^a–c^ Values (mean ± SD, from three replicate cheese-making trials) in each row with different letters were different significantly (*p* < 0.05). AR, the area ratio of volatile and internal standard (IS).

#### 2.2.1. Ketones

For many cheeses, methyl ketones are by far the most abundant compounds. The two major methyl ketones are heptan-2-one and nonan-2-one, considering the quantity of octanoic and decanoic acids present in milk fat [22]. In our research, all ketones detected were methyl ketones from C3 to C11 including acetoin, acetophenone and 3,5-octadien-2-one, in addition to 5 alkan-2-ones and two diketones. 

The cheese made with *S. thermophilus* SP1.1 showed lower contents of heptan-2-one and nonan-2-one than the other two cheeses (*p* < 0.05). The results suggested that *S. thermophilus* TM11 and SP1.1 had different synthesis and degradation abilities for heptan-2-one and nonan-2-one. Two metabolism pathways of methyl ketones have been certified: the degradation of the corresponding fatty acid and the successive β-oxidation of long-chain fatty acid [23], both of which can be performed by the enzymes from bacteria. 

The presence of acetone was observed in CRMP, and a decreased level was found in the cheese made with *S. thermophilus* SP 1.1 compared to other cheeses (*p* < 0.05). We also identified relatively high levels of acetophenone and 3,5-octadien-2-one, especially in the cheese with *S. thermophilus* TM11 (AR > 1).

The production of diacetyl and acetoin, obtained from pyruvate, is mainly due to the activity of lactic acid bacteria, especially *Lactococcus lactis* ssp. *lactis* biovar *diacetylactis*. Diacetyl seems to be produced in lesser amounts than acetoin and can be reduced to acetoin by the action of a diacetyl reductase in some bacteria [24,25]. In our study, however, higher contents of 2,3-butanedione than acetoin were displayed both in the cheeses made with *S. thermophilus* TM11 and SP1.1 (Table 2). The result might be due to the differences of some reductase in *S. thermophilus* TM11 and SP1.1, such as the reductases of diacetyl or acetoin. Moreover, this result might be attributed to the heat history of the milk powder used. Yuceer *et al.* [4] identified diacetyl as a heat-generated aroma compound in Ezine cheeses and did not find acetoin.

#### 2.2.2. Aldehydes

Eight aldehydes from C6 to C10 were found in our research, including five alkyl, two alkenyl and one phenyl aldehyde (Table 2). Compared to DAC, the cheese made with *S. thermophilus* SP1.1 had the lower contents of heptanal and nonanal, while the cheese made with *S. thermophilus* TM11 had higher contents of hexanal, 2-hexenal, benzaldehyde and octanal (*p* < 0.05). The contents of 2-nonenal and decanal in the three different cheese varieties were not significantly different (*p* > 0.05). Hexanal, a grass aroma, was an important contributor of flavor in some famous cheeses [4,26,27]. Other compounds of this group were also identified in many cheeses.

Furthermore, branched-chain aldehydes were not found in CRMP. These compounds derived from branched-chain amino acids have been identified in many cheeses [28,29,30]. It is well known that 3-methylbutanal is considered the major contributor to malty flavors in milk and harsh flavors in cheeses. 

#### 2.2.3. Acids, Alcohols and Esters

Butyric and hexanoic acids are common odor-active free fatty acids contained in cheeses [31]. Higher contents of butyric, hexanoic and octanoic acids were found in CRMP with *S. thermophilus* SP1.1 or TM11 than DAC in our research. Use of *S. thermophilus* TM11 significantly increased (*p* < 0.05) the contents of butyric and hexanoic acids than SP1.1.

In addition, alcohols were found in most of cheese varieties, and are originated from the fermentation of lactose by heterofermentative LAB [32], from amino acid metabolism [33], or from aldehyde reduction [22]. These compounds, which are responsible for the strong and exciting flavors in cheese, were not detected in CRMP. Furthermore, we did not find the branched-chain alcohols and esters which are common in cheeses [28,29] and are usually regarded as part of the undesirable aroma [34,35].

Except for the branched-chain esters, four unbranched esters and a branched-chain unsaturated ester (methyl 2-methyl-2-propenoate) were found in CRMP with the high AR values, but they were not statistically significant among the different CRMP varieties. The reason may be due to the heat history of milk powder and the use of flavoring agents. Although some authors considered that esters were produced by the organism, e.g., wild *Lactococcus lactis* strains [30], esters can also be formed during the extraction of the culture distillate or concentration of the extract [36]. Besides, methyl 2-methyl-2-propenoate is commonly used in milk products as a flavoring agent, and no references reported it as a milk component or a compound in milk products.

#### 2.2.4. Sulfur-Containing Compounds

Sulfur-containing compounds, including thiourea and CS_2_, were identified in our study, and showed no changes among the cheese varieties. Although its origins are still unknown, CS_2_ has been identified in many cheese varieties [37,38,39] as a breakdown product of other sulfur compounds [35]. CS_2_ was also found in stored milk powder [40], heated milk and even fresh milk [41].

#### 2.2.5. Other Volatiles

This group of volatiles includes the benzenic compounds (benzene, toluene, ethylbenzene and xylene), indene, ethoxyphenyl oxime and limonene. Most of these volatiles had relatively high contents, except for benzene (AR < 0.1). Benzenic compounds were found in many cheese varieties. Centeno *et al.* [30] considered toluene to be significantly influenced by strains of *Lactococcus lactis*. In our study, addition of *S. thermophilus* SP1.1 significantly decreased the contents of ethylbenzene and xylene as well as indene, in contrast to the other cheeses, whereas, the cheese made with *S. thermophilus* TM11 had the highest content of toluene (*p* < 0.05). Limonene, a terpene that stems from forage, was commonly identified in cheeses [42], and showed no significantly change among different CRMP varieties in our study. There is little information on methoxyphenyl oxime, but the compound has been found in milk [43] and cheese products [37]. CRMP made with *S. thermophilus* SP1.1 had a higher content of methoxyphenyl oxime than other cheeses, whereas, there was an absence of this compound in DAC, suggesting that it might be produced by bacteria.

### 2.3. Texture of CRMP

#### 2.3.1. The Effect of Storage Time

**Figure 2:**
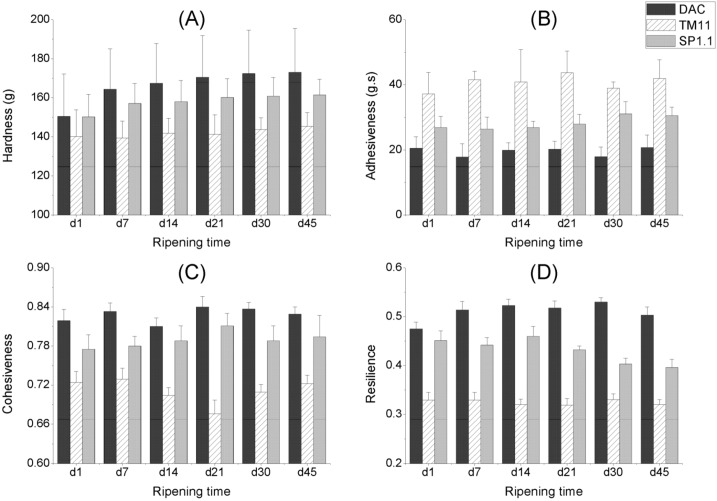
Changes in textural properties of the direct-acidified cheese (DAC) and the cultured cheeses with *S. thermophilus* (TM11 and SP1.1) during storage.

The textural properties of CRMP are shown in Figure 2. The variables, including hardness, adhesiveness, cohesiveness and resilience, were acquired to assess the effect of *S. thermophilus* on cheese texture. Although the adhesiveness, cohesiveness and resilience did not show a clear trend, the hardness of DAC and the cheese made from *S. thermophilus* SP1.1 increased with increasing cheese storage time within each cheese trial. However, no significant (*p* > 0.05) differences in this texture variable were observed among the different storage days within the three cheese trials. The results indicated that such storage (6 °C for 45 days) would not change the texture of any cheese varieties. This finding might be due to mild degradation of proteins because the textural changes during cheese ripening could be attributed to the proteolysis of cheeses [44], which leads to decreases in hardness of the cheese texture [45].

#### 2.3.2. The Effect of Starter Cultures

Differences were found in the mean values of textural variables (Figure 2). The use of *S. thermophilus* TM11 generally resulted in cheeses with the lowest hardness, cohesiveness, resilience and the highest adhesiveness, whereas DAC showed the opposite results, regardless of the storage time. However, the changes in hardness are not significant (*p* > 0.05) among the different CRMP, regardless of the storage time within the three cheese trials.

No significant differences in adhesiveness were observed among the different CRMP in the first day, but the cheese made with *S. thermophilus* TM11 showed significantly increased adhesiveness in the other days (*p* < 0.05) compared to DAC. Moreover, *S. thermophilus* TM11 led to cheeses with lower cohesiveness and resilience than DAC, regardless of the storage time. The cheese made with *S. thermophilus* SP1.1 did not display textural variable changes, except that resilience in days 7 to 45 showed a slightly decrease, in contrast to DAC.

If a food has lower adhesiveness compared with cohesiveness, then the probe is likely to remain clean [46], *i.e.*, the food has the ability to hold together. The ratios of adhesiveness to cohesiveness ranged in a diminishing sequence from *S. thermophilus* TM11 (50.7–64.0, 95% confidence interval for mean) to SP1.1 (32.8–39.0) to DAC (20.6–26.6), and showed a significant difference (*p* < 0.01) regardless of the storage time.

Texture is one of the most important characteristics of cheese that determines identity and acceptability [44]. Lowfat cheese has a rubbery, dry and hard body and texture [15]. The use of some special cultures can improve the body and texture of the low fat cheese due to the high proteolysis levels [8], but proteolysis may lead to strong and exciting flavors which further result in low acceptability for some consumers. In the research, EPS-producing *S. thermophilus* TM11 improved the texture properties by increasing the moisture retention as well as by producing the proper flavors in cheese.

### 2.4. Microstructure of CRMP

A changed moisture or fat content would affect the cheese microstructure [21]. To evaluate the effect of the EPS on the microstructure of CRMP, scanning electron microscopy was performed. This technique allows the preservation of the original structure of the sample as far as possible because of the gradual replacement of the water by ethanol.

**Figure 3:**
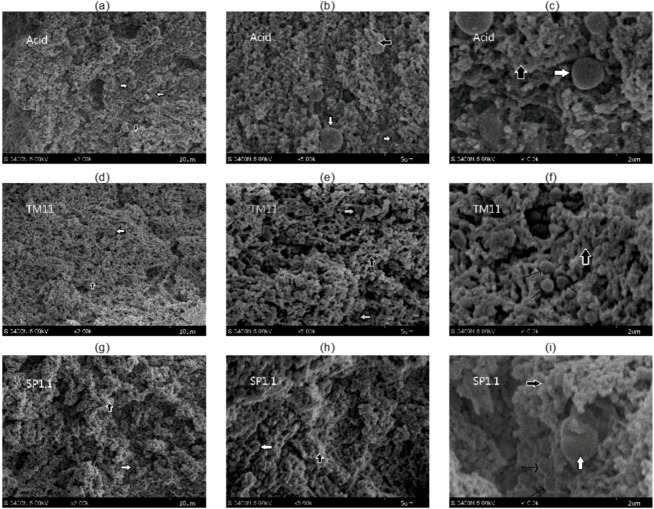
Microstructures of the direct-acidified cheese (DAC) and the cultured cheeses with *S. thermophilus* (TM11 and SP1.1). The white thick arrow points to fat globules. The black thick arrow points to the protein matrix. The thin arrow points to the bacteria.

The microscopic studies of three different amplifications of CRMP were demonstrated in Figure 3. DAC (Figure 3–c) displayed its protein matrix (black thick arrow) formed by casein micelles. Fat globules (white thick arrow) were largely spherical in shape with the diameter of ~1.5 μm, and were present in the matrix of proteins. Fat globules, as well as water (pockets in microscope), can function as casein networks breakers [44,47]. However, the water in large pockets would lose its stability under pressure or long storage causing the dehydration of cheese.

Although *S. thermophilus* SP1.1 (Figure 3g,h,i) did not obviously affect the microstructure, the use of *S. thermophilus* TM11 (Figure 3d,e,f) in CRMP greatly changed the protein matrix with more opened cavities. The cavities were numerous and small. These results agreed with the reported studies in which a more open structure was observed in Mozzarella [9], Feta [48] and Panela [21] cheese made with the EPS-forming culture of *S. thermophilus*. Accordingly, these cavities could contain more water, which was in agreement with the increasing moisture and yield of CRMP.

## 3. Experimental

### 3.1. Cheese Manufacture

At each treatment, 3 L 12% (w/v) reconstituted part-skim milk powder was prepared at a protein-to-fat ratio of 1.6 with a medium-heat whole milk powder and a low-heat skim milk powder (Nestle, Heilongjiang, China) according the content of milk components in their product descriptions. The milk was agitated for 30 min at ~600 rpm and then heated at 80 °C for 5 min (excluding the time of heating and cooling) for sterilization in a stainless vat (20 cm × 25 cm with a height of 20 cm).

*S. thermophilus* TM11 and SP1.1 were stored in a −80 °C refrigerator in the skim milk-based medium. These frozen cultures were transferred to M17 broth supplemented with 0.5% (w/v lactose (LM17), and then cultured at 37 °C for activation. The last culture was inoculated in the reconstituted part-skim milk powder (100 mL each) described above. These cultures were stored at 4 °C for use in the next day.

The cheese manufacturing was performed according to Perry *et al*. [9] with the following modifications: three vats were used each time with different treatments in a water bath of 37 °C. The milk in vats 1 (control) was inoculated with no starter, but acidified to pH 6.0 using lactic acid diluted 1:4 (v/v) with distilled water. The milk in vats 2 and 3 was inoculated with the prepared cultures of *S. thermophilus* TM11 and SP1.1, respectively. The inocula (3%, v/v) of starter culture were determined according to the preliminary experiments, and the time and endpoint pH of fermentation. After pH in vats 2 and 3 reached 6.2 (40–50 min of ripening), commercial calf rennet (Naturen, Chr. Hansen’s Laboratory, Copenhagen, Denmark) was added to reach a final concentration of 0.2 g/L as well as vats 1. The amount of rennet added was selected according to manufacturing times corresponding to industry practices. After final drain, the curd was salted by dry-stirring 2.0% (w/w) salt in each vat. The curd was hand-stretched for 5 min at 65 °C. Three independent replicates of cheese manufacturing were performed in different days.

### 3.2. Physicochemical and Microbiological Analyses

The yield of cheese was analyzed as the ratio (%) of the weight of cheese and the weight of cheese milk at first day of storage. Each cheese was divided into six parts and sealed in plastic bags. These cheeses were stored at 6 °C for analysis in different ripening days. Moisture (%) was determined according to the weight loss by drying sample at 105 ± 1 °C. The salt content was determined by the Volhard method [49]. Protein was determined by Kjeldahl method [49,50] with conversion to protein content using a factor of 6.38. The pH was monitored with a combined electrode pH-meter (PB-10, Sartorius, Gottingen, Germany). A sample of cheese (10 g), was aseptically transferred to sterile flask and homogenized with 0.9% (w/v) sterile saline water (90 mL) for 1 min at room temperature. Serial decimal dilutions were prepared with the sterile saline water for estimating the counts of lactococci, which were inoculated on LM17 media at 37 °C for 72 h.

### 3.3. Volatile Compounds

For the extraction of volatile compounds, grated cheese (30 g) and NaH_2_PO_4_ (30 mL, 25%, w/v) were added in a 100 mL flask equipped with a stirring bar. An aqueous solution of 4-methyl-2-pentanone (50 mg/L, 0.2 mL) was added as internal standard. The sealed vial was equilibrated for 30 min at 50 °C in a thermostatic bath. A 2 cm 50/30 μm fiber (DVB/CAR/PDMS, Supelco, Bellefonte, PA, USA) equipped with a SPME manual holder (Supelco) was desorbed at 250 °C for 2 min and then inserted in the vial to extract the volatiles for 45 min. The equilibration and extraction were stirring with a magnetic stirrer (HJ-3, Guohua Inc., Jiangshu, China).

The volatiles isolated by the fiber were desorbed in the injector port of a GC (Hewlett-Packard 6890, Wilmington, DE, USA) equipped with the HP 5973 mass selective detector (Hewlett-Packard Inc.). A DB-5 capillary column with 60 m length, 0.25 mm internal diameter, 0.25 μm phase thickness (J&W Scientific, Folsom, CA, USA) was used. The oven temperature was held at 40 °C for 8 min, increased from 40 °C to 150 °C at the rate of 4 °C/min and from 150 to 250 °C at 20 °C/min, then hold at 250 °C for 5 min. The temperatures of the injector and detector were 250 and 280 °C, respectively. The flow rate of helium carrier gas was 1.0 mL/min. Electron impact ionization was used at a voltage of 70 eV. The mass range was *m/z* 30–500. The relative levels of individual compounds were assessed using the AR value treated as the area ratio of volatile and internal standard. 

### 3.4. Textural Profile Analysis (TPA) of Cheese

The compression testing was performed on the cheeses after 1, 7, 14, 21, 30 and 45 days of ripening using a texture analyzer (TA.XT.plus, Stable Micro Systems Ltd., Surrey, UK) equipped with a 5 kg load cell. A plunger, 35 mm in diameter, was attached to the moving crosshead. Cubes (1 cm^3^) from each cheese were prepared and allowed to equilibrate to assay temperature (20 ± 1 °C). The operating conditions were: test speed of 60 mm/min and two compression mode. From each force-time curve, obtained by compression of the sample to 75% of its original height, the following texture-profile parameters were determined: (1) hardness, the compressive force (g) recorded at maximum compression, *i.e.*, the force recorded at 75% compression of the sample; (2) adhesiveness, the negative force area (g.s) during the first compression; (3) cohesiveness, the ratio of the positive force area during the second compression to that during the first compression; and (4) resilience, the ratio of the positive force area during down-compression to that during up-compression in the first circle. At least six replicate measurements were made for each sample and the average value (±S.E.) for the three cheese-making trials was reported.

### 3.5. Microstructure

Samples of cheese were cut into pieces (2 mm × 2 mm with a height of 0.5 mm) using a sterile blade, were immersed in 2% glutaraldehyde, and were stored at 4 °C. The cheese samples were then prepared for scanning electron microscopy using the method of Serrano *et al*. [51].

### 3.6. Statistical Analysis

All the experiments mentioned above were performed in triplicate in different days. Experimental data were statistically analyzed using PASWStatistics18.0 Software (SPSS Inc, Chicago, IL, USA). One-way analysis of variance (ANOVA) using the least significant difference (LSD) test (*p* < 0.05) was applied to examine the effect of different treatments.

## 4. Conclusions

The results of this study indicated that the use of direct acidified CRMP resulted in a decrease of moisture content from 65.0% to 62.5% to 59.9% on days 1, 21 and 45 of storage, respectively. Use of *S. thermophilus* TM11 could hold the moisture of cheese, and consequently showed an increase in the yield (by 4.6%) and a decrease of protein content (by 6.6%), compared to the direct acidified cheese.

The major volatiles in CRMP included aldehydes, ketones and acids. The esters and sulfur compounds that are produced from the heating and drying treatments of milk had an important effect on the flavor of CRMP. Alcohols and the branched-chain aldehydes that contribute to the exciting and harsh flavors of cheese were not found. The cheese made with *S. thermophilus* TM11 had lower cohesiveness, resilience and higher adhesiveness than DAC, and had similar hardness. In the meantime, *S. thermophilus* TM11 in CRMP greatly changed the protein matrix to a porous network with more opened cavities, indicating that the changes in microstructure were responsible for the differences in the texture and technical properties.
